# Bioenergetic Signatures of DLD Deficiency: Dissecting PDHc- and α-KGDHc-Linked Defects

**DOI:** 10.3390/antiox15010019

**Published:** 2025-12-22

**Authors:** Yarden Haham Zarbib, Shira Huri Ohev-Shalom, Shani Kassia Lyskov, Yuval Mazor, Mika Anekstein-Spigel, Nechama Shalva, Ronen Spiegel, Orna Staretz-Chacham, Joshua Manor, Ann Saada, Rachel Rock, Yair Anikster, Tal Yardeni

**Affiliations:** 1 Metabolic Center, Sheba Medical Center, Tel-Hashomer, Ramat Gan 52621, Israel; yarden.haham@sheba.health.gov.il (Y.H.Z.); shira.huriohevshalom@sheba.health.gov.il (S.H.O.-S.); shanilyskov@mail.tau.ac.il (S.K.L.); yuval.mazor@sheba.health.gov.il (Y.M.); mika.aneksteinspigel@sheba.health.gov.il (M.A.-S.); 2 Faculty of Medicine and Life Sciences, Tel Aviv University, Tel Aviv 69978, Israel; yehoshua.manor@sheba.health.gov.il (J.M.); yair.anikster@sheba.health.gov.il (Y.A.); 3 Metabolic Disease Unit, Edmond and Lily Safra Children’s Hospital, Sheba Medical Center, Ramat Gan 52621, Israel; nechama.shalva@sheba.health.gov.il (N.S.); rachel.rock@sheba.health.gov.il (R.R.); 4 Department of Pediatrics B, Emek Medical Center, Afula 23100, Israel; spiegel_ro@clalit.org.il; 5 Rappaport Faculty of Medicine, Technion, Haifa 3525433, Israel; 6 Metabolic Clinic, Pediatric Division, Soroka University Medical Center, Ben Gurion University, Beer Sheva 84105, Israel; staretz@bgu.ac.il; 7 Department of Genetics, Hadassah Medical Center and Faculty of Medicine, Hebrew University of Jerusalem, Jerusalem 91220, Israel; ann.saadareisch@mail.huji.ac.il; 8 Department of Medical Laboratory Sciences, Jerusalem Multidisciplinary College, Jerusalem 9422408, Israel

**Keywords:** dihydrolipoamide dehydrogenase deficiency, pyruvate dehydrogenase complex, α-ketoglutarate dehydrogenase complex, mitochondrial bioenergetics, complex I dysfunction, high-resolution respirometry, diagnostic assay

## Abstract

Dihydrolipoamide dehydrogenase (DLD) deficiency (MIM #246900) is a rare autosomal recessive mitochondrial disorder caused by pathogenic variants in the *DLD* gene, which encodes the E3 subunit common to multiple mitochondrial enzyme complexes, including pyruvate dehydrogenase (PDHc) and α-ketoglutarate dehydrogenase (αKGDHc). Although genotype–phenotype correlations have been described, the precise bioenergetic consequences of DLD dysfunction remain poorly defined. Here, we applied high-resolution respirometry using a novel single-run protocol that allows simultaneous assessment of mitochondrial respiratory capacity and, critically, distinguishing between PDHc- and αKGDHc-linked respiration within the same assay. Fibroblasts from six genetically confirmed DLD-deficient patients with distinct pathogenic variants and clinical severities exhibited a consistent reduction in maximal and complex I-linked respiration. The most severe cases (c.1436A>T; p.D479V) showed combined PDHc and αKGDHc impairment, whereas milder genotypes displayed isolated PDHc dysfunction. This mechanistic distinction likely underlies the variable clinical response to ketogenic therapy, which depends on intact αKGDHc function. Analysis of the mitochondrial mass and mtDNA copy number revealed no global reduction, indicating intrinsic enzymatic dysfunction as the primary defect. Collectively, this study defines a reproducible bioenergetic signature of DLD deficiency and introduces an integrated one-run diagnostic strategy for delineating enzyme-specific mitochondrial defects, providing a framework for mechanistic and therapeutic investigations.

## 1. Introduction

Dihydrolipoamide dehydrogenase (DLD, lipoamide dehydrogenase) deficiency (MIM #246900) is a rare autosomal recessive mitochondrial disorder caused by pathogenic variants in the *DLD* gene, which encodes the flavoprotein DLD, also known as the E3 component of mitochondrial dehydrogenase complexes [1]. DLD serves as the E3 subunit in three key mitochondrial multienzyme complexes: the pyruvate dehydrogenase complex (PDHc), α-ketoglutarate dehydrogenase complex (αKGDHc), and branched-chain α-keto acid dehydrogenase complex (BCKDHc); it also participates in the glycine cleavage system [2,3,4]. Through its role in oxidizing dihydrolipoamide to lipoamide, DLD is essential for oxidative decarboxylation of pyruvate, α-ketoglutarate, and branched-chain amino acids, thereby linking carbohydrate, amino acid, and energy metabolism [2,5,6].

**Figure 1:**
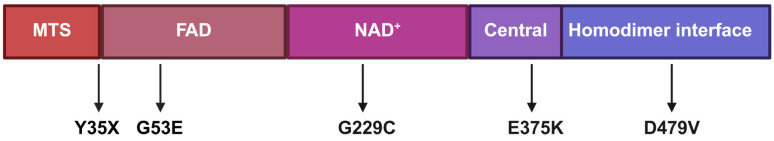
Schematic representation of *DLD* mutations identified in the patient cohort. The positions of the pathogenic variants included in this study (Y35X—c.105insA; p.Y35X, G53E—c.158G>A; p.G53E, G229C—c.685G>T; p.G229C, E375K—c.1123G>A; p.E375K, and D479V—c.1436A>T; p.D479V) are mapped along the DLD protein structure. The domain organization includes the mitochondrial targeting sequence (MTS), flavin adenine dinucleotide-binding domain (FAD), NAD^+^-binding domain (NAD^+^), central domain, and homodimer interface. Only the variants identified in the six DLD-deficient patients analyzed in this study are depicted. The mutations localize to distinct structural domains of the enzyme, potentially affecting cofactor binding, dimerization, or catalytic activity, and may underlie the observed variability in clinical severity and mitochondrial respiration profiles. Created in BioRender. Yardeni, T. (2025) https://BioRender.com/qxf0i7r.

Pathogenic variants in the human *DLD* gene have been identified mainly in three structural regions (Figure 1): the homodimer interface (e.g., c.1436A>T; p.D479V) [5,7,8], the disulfide-exchange (active) site, and the FAD/NAD^+^ binding sites (e.g., c.685G>T; p.G229C) [3,5,8,9]. Variants in the dimer interface likely impair protein homodimerization, resulting in reduced enzymatic activity, while substitutions near the active site are also predicted to disrupt catalytic activities [5,7]. The c.685G>T; p.G229C variant, an Ashkenazi Jewish founder mutation, is most often associated with a predominantly hepatic phenotype with neurological involvement [3,9], whereas the c.1436A>T; p.D479V variant is linked to a severe neurodegenerative presentation [7,8].

From a clinical point of view, DLD deficiency exhibits striking phenotypic variability, with manifestations that may involve the nervous system, liver, and muscle [1,3,4]. Reported features include recurrent vomiting, episodic encephalopathy, elevated liver transaminases, hypoglycemia, developmental delay, lactic acidosis, and infantile spasms [1,3,4,9]. The disorder’s heterogeneity likely reflects both the structural consequences of individual variants and the degree of residual enzyme activity. In addition, the ATP production rate had a direct relationship with the severity of the neurological involvement [10].

While previous studies have reported genotype–phenotype correlations, biochemical findings, and enzymatic activity in patient-derived samples [1,3,4,9,11], functional profiling of the mitochondrial respiratory chain in DLD deficiency has generally been performed in a fragmented manner using several separate assays. Notably, most previous studies did not distinguish between respiratory defects arising from dysfunction of the PDHc or αKGDHc complexes, a distinction that is crucial for elucidating the biochemical basis of DLD deficiency. This limitation likely reflects the technical challenges of accurately assessing the activity of these mitochondrial multienzyme complexes using traditional radiochemical assays [12], which require separate, labor-intensive measurements under highly controlled conditions.

In this study, we examined fibroblasts from six genetically confirmed DLD-deficient patients carrying different mutations and presenting with varying clinical severities. Using high-resolution respirometry, we demonstrated an overall decrease in mitochondrial respiration in a single experimental regime, including quantification of the relative contribution of both complex I and complex II-dependent respiration. This provides a comprehensive view of the specific bioenergetic defects associated with each patient’s genotype. Importantly, this integrated approach also enabled us to distinguish between PDHc- and αKGDHc-linked respiration, offering novel mechanistic insights into the enzymatic basis of DLD deficiency. This strategy allowed us to generate detailed respiratory profiles and assess complex-specific contributions, thereby providing novel biochemical insights into the variable pathophysiology of DLD deficiency.

## 2. Materials and Methods

### 2.1. Samples

Dermal fibroblast primary cell lines from six genetically confirmed patients with DLD deficiency were obtained from the Pediatric Metabolic Disease Unit, Sheba Medical Center (IRB# SMC-21-8644, Figure 1, Table 1, and Supplementary Figure S1), as well as two control cell lines: a control human dermal fibroblast cell line was purchased from ATCC (PCS-201-012; Ctrl 1, Manassas, VA, USA), and a primary cell line from a 39-year-old healthy male (Ctrl 2). Cells were maintained in culture for no more than 10 passages prior to experiments.

Dermal fibroblast cell lines (patient-derived and control) were thawed and cultured in high-glucose DMEM (4.5 g/L D-glucose; Gibco, Thermo Fisher Scientific Inc. Waltham, MA, USA) supplemented with 20% heat-inactivated fetal bovine serum (FBS; Gibco, Thermo Fisher Scientific Inc. Waltham, MA, USA), penicillin-streptomycin (10,000 U/mL and 10,000 µg/mL, respectively, Gibco, Thermo Fisher Scientific Inc. Waltham, MA, USA), sodium pyruvate (1 mM, Gibco, Thermo Fisher Scientific Inc. Waltham, MA, USA), and L-glutamine (2 mM, Gibco, Thermo Fisher Scientific Inc. Waltham, MA, USA). Cultures were maintained at 37 °C in 5% CO_2_. For all experiments, 24 h prior to assays, the medium was switched to low-glucose (regular) DMEM (1 g/L D-glucose; Gibco, Thermo Fisher Scientific Inc. Waltham, MA, USA) supplemented identically as described above. High-glucose medium was used for routine fibroblast expansion, while the 24 h shift to low (regular)-glucose medium reduces PDH E1α phosphorylation via PDKs [13], ensuring more consistent PDHc activation across lines. Additionally, lowering glucose availability forces the cells to rely more heavily on mitochondrial oxidative metabolism, which enhances the sensitivity of downstream respirometry measurements. For the DLD activity assay, cells were thawed and cultured only in high-glucose DMEM.

### 2.2. Structural Modeling of DLD Variants

A three-dimensional structural model of the human DLD protein was obtained from the AlphaFold Protein Structure Database. The predicted structure for DLD (UniProt P09622) was retrieved from the AlphaFold server (https://alphafold.ebi.ac.uk/entry/AF-P09622-F1, accessed on 24 November 2025), and patient-derived variants (c.105insA; p.Y35X, c.158G>A; p.G53E, c.685G>T; p.G229C, c.1123G>A; p.E375K, c.1436A>T; p.D479V) were mapped onto the structure for visualization.

### 2.3. High-Resolution Respirometry

**Figure 2:**
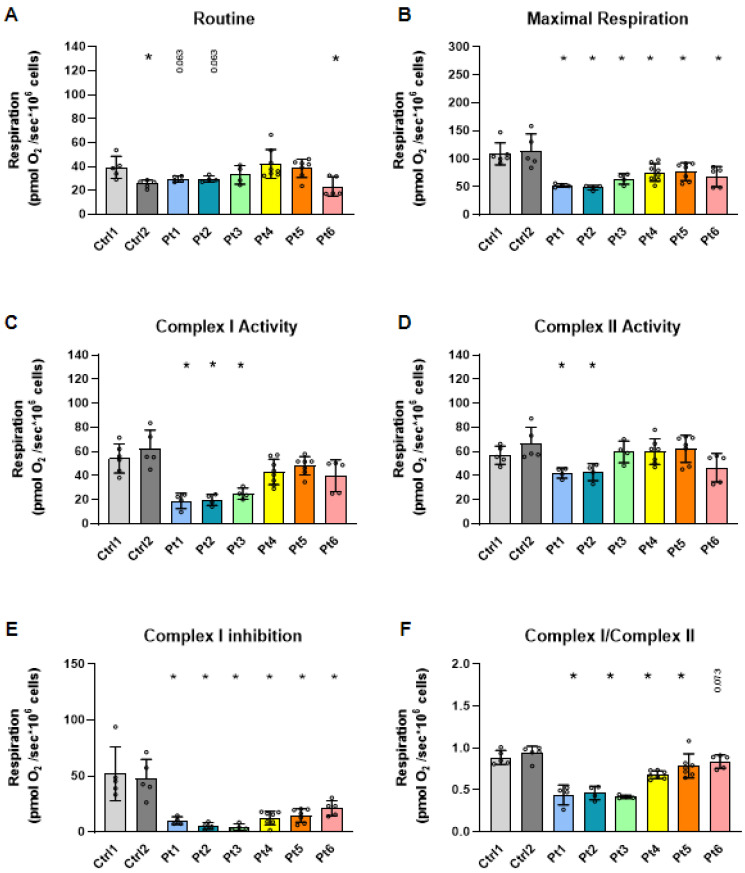
Mitochondrial respiration is impaired in fibroblasts derived from patients with DLD deficiency. Mitochondrial oxygen consumption was assessed in controls (Ctrl1 and Ctrl2) and patient (Pt1–Pt6) fibroblasts using high-resolution respirometry (Oroboros O2k). (**A**) Routine respiration; (**B**) maximal respiration calculated as the difference between FCCP-stimulated and α-chaconine–permeabilized rates; (**C**) complex I-linked respiration (NADH-linked respiration, N-pathway), calculated as the difference between ADP and α-chaconine; (**D**) complex II-linked respiration (NS-pathway) calculated as the difference between respiration after rotenone and α-chaconine addition; (**E**) effect of complex I inhibition, calculated as the difference between FCCP-stimulated and rotenone-inhibited respiration; and (**F**) complex I/complex II respiration ratio (complex I-linked activity divided by complex II-linked activity). Each open circle represents an independent experimental run (N = 4–8 repeats per sample). All data were normalized to cell number. Statistical analysis was performed using the Mann–Whitney U test. * *p* < 0.05 vs. Ctrl1; numerical *p* values (0.05 < *p* < 0.1) are indicated on the plots. Abbreviations: Ctrl, control; Pt, patient.

**Figure 3:**
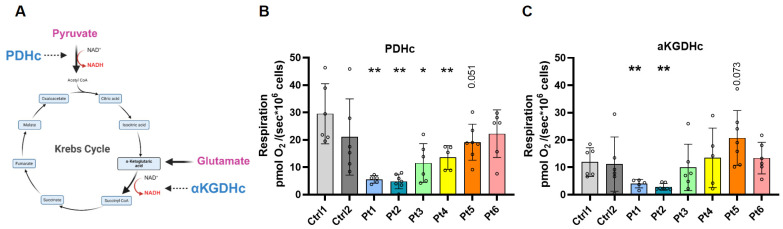
Effects of *DLD* mutations on mitochondrial enzymes containing the E3 subunit. (**A**) Schematic representation of mitochondrial dehydrogenase complexes incorporating the DLD (E3) subunit. The addition of pyruvate reflects pyruvate dehydrogenase complex (PDHc)-linked activity, whereas the subsequent addition of glutamate represents α-ketoglutarate dehydrogenase complex (αKGDHc)-linked respiration. (**B**) PDHc activity, calculated by the increase in oxygen flux following the addition of ADP after pyruvate supplementation. (**C**) αKGDHc activity, calculated by the increase in oxygen flux following the addition of glutamate after ADP supplementation. Each open circle represents an independent experimental run (N = 5–7 repeats per sample). The data were normalized to cell number, and statistical analysis was performed using the Mann–Whitney U test (* *p* < 0.05, ** *p* < 0.01 vs. Ctrl 1); numerical *p* values (0.05 < *p* < 0.1) are indicated on the plots. Abbreviations: Ctrl, control; Pt, patient. Panel A created in BioRender. Yardeni, T. (2025) https://BioRender.com/17nt3qt.

Mitochondrial respiration was assessed using the O2k-FluoRespirometer (Oroboros Instruments, Innsbruck, Austria) by standard substrate–uncoupler–inhibitor titration (SUIT) protocols for OXPHOS analysis. Briefly, fibroblasts (0.7–1 × 10^6^ cells per chamber) from an 80% confluent T75 flask were harvested after 24 h incubation in low-glucose medium and transferred into chambers containing mitochondrial respiration medium (MiR05). Routine respiration, defined as oxygen consumption in intact fibroblasts in assay medium without pharmacological intervention, was recorded prior to permeabilization. After this initial measurement, either digitonin (8 µM, Sigma-Aldrich, St. Louis, MO, USA) or α-chaconine (20 µM; PhytoLab GmbH & Co. KG, Vestenbergsgreuth, Germany) was applied to selectively permeabilize the plasma membrane. For each patient line, 0–3 independent experiments were performed using digitonin and 2–8 using α-chaconine (Figure 2), whereas all experiments shown in Figure 3 were conducted with α-chaconine. As expected based on the literature, we observed no differences in mitochondrial integrity or respiratory performance between the two permeabilization methods [14]. Oxygen flux was recorded in real time under the following sequential substrate and inhibitor additions: Pyruvate (5 mM; Sigma-Aldrich, St. Louis, MO, USA), and malate (2 mM; Sigma-Aldrich, St. Louis, MO, USA) were added to initiate electron entry through complex I [NADH-linked respiration (N-pathway substrates)], followed by ADP (2.5 mM; Merck KGaA, Darmstadt, Germany) to activate OXPHOS capacity (N-pathway); succinate (10 mM; Sigma-Aldrich, St. Louis, MO, USA) was supplied to stimulate complex II-linked respiration, enabling assessment of OXPHOS capacity through convergent electron flow via complexes I and II (NS-pathway). Cytochrome c (10 µM; Sigma-Aldrich, St. Louis, MO, USA) was then introduced as a control for mitochondrial outer membrane integrity. Oligomycin (5 nM; Sigma-Aldrich, St. Louis, MO, USA) was added to inhibit ATP synthase and determine proton leak–driven respiration, and FCCP (Sigma-Aldrich, St. Louis, MO, USA) was titrated stepwise to a maximum of 4 µM to uncouple respiration and measure maximal electron transport system (ETS) capacity. Finally, rotenone (0.5 µM; Sigma-Aldrich, St. Louis, MO, USA) was added to inhibit complex I, thereby enabling evaluation of complex II activity, followed by antimycin A (2.5 µM; Sigma-Aldrich, St. Louis, MO, USA) to inhibit complex III and quantify residual non-mitochondrial oxygen consumption. After each addition, a steady-state plateau was confirmed prior to the next titration. Chambers were reoxygenated by briefly opening the stoppers for ~5 min following oligomycin addition. Data acquisition and analysis were performed using DatLab 7 software (Oroboros Instruments, Innsbruck, Austria). Whenever feasible, patient and control fibroblasts were assayed simultaneously in parallel chambers of the same O2k instrument to minimize inter-assay variability. In addition, the instrument was calibrated to room oxygen within 24 h prior to each experiment, further reducing day-to-day variation.

### 2.4. Approach for Discriminating PDHc- Versus αKGDHc-Linked Respiratory Pathways

To differentiate between the contributions of the PDHc and αKGDHc to mitochondrial respiration, we developed a modified SUIT protocol. Fibroblasts (0.7–1 × 10^6^ cells per chamber) from an 80% confluent T75 flask were harvested after 24 h in low-glucose medium, transferred into MiR05, and permeabilized with α-chaconine (20 μM). Oxygen flux was then recorded under the following sequential substrate additions: pyruvate (5 mM) to initiate PDHc-dependent respiration, followed by ADP (2.5 mM) to stimulate OXPHOS capacity. Glutamate (10 mM; Sigma-Aldrich, St. Louis, MO, USA) was subsequently added to evaluate αKGDHc-linked respiration. Then, malate (2 mM) was provided to sustain NADH-linked flux. Critically, αKGDHc-linked respiration was quantified immediately after the addition of glutamate, whereas malate was introduced only afterward to support downstream TCA-cycle flux without contributing to the αKGDHc measurement itself. The remainder of the protocol followed standard titrations: succinate (10 mM) for complex II respiration, cytochrome c (10 µM) was used to verify mitochondrial outer membrane integrity, oligomycin (5 nM) for ATP synthase inhibition, FCCP (up to 4 µM) for maximal ETS capacity, and the sequential addition of rotenone (0.5 µM) and antimycin A (2.5 µM) to inhibit complexes I and III, respectively, enabling quantification of residual non-mitochondrial respiration. Data acquisition and analysis were performed using DatLab 7 software (Oroboros Instruments).

### 2.5. DLD Enzymatic Activity Assay

Fibroblast pellets were collected, washed in PBS, and stored dry at −80 °C until analysis. For the DLD (LAD) enzymatic assay, frozen fibroblasts were resuspended, sonicated, and lysed in assay buffer containing Triton X-100, and DLD activity was measured spectrophotometrically by monitoring NADH oxidation in the presence of lipoamide, as previously described [10]. Enzymatic activity was calculated from the linear rate of absorbance change and expressed as mU/mg total protein. Total protein content for normalization was determined using the Lowry method with bovine serum albumin as the standard, as described in the original protocol [10]. Chemicals and reagents were purchased from Sigma-Aldrich, St. Louis, MO, USA.

### 2.6. Mitochondrial DNA (mtDNA) Copy Number

The mtDNA copy number in fibroblasts was determined as previously described [15]. DNA was extracted using the DNeasy Blood & Tissue Kit (QIAGEN, Hilden, Germany) according to the manufacturer’s instructions. Quantitative real-time PCR (qPCR) was performed with 60 ng input DNA per reaction using Fast SYBR™ Green Master Mix (Thermo Fisher Scientific, 4385612, Waltham, MA, USA). Amplification targeted the mitochondrial gene: *tRNA^Leu^* (F-CACCCAAGAACAGGGTTTGT R-TGGCCATGGGTATGTTGTTA) (MT-TL1), with the nuclear-encoded gene (nDNA) *B2-microglobulin* (F-TGCTGTCTCCATGTTTGATGTATCT R-TCT CTGCTCCCCACCTCTAAGT) (NM_004048) serving as the reference. Cycle threshold (CT) values were used to calculate relative mtDNA copy number according to: ΔCT = (CT_nDNA − CT_mtDNA) and then relative mtDNA copy number = 2 × 2^ΔCT^.

### 2.7. Flow Cytometry Analysis of Mitochondrial Content

Mitochondrial content was assessed in fibroblast cell lines using MitoTracker Green (MTG; Thermo Fisher Scientific, Waltham, MA, USA). Cells (~3–5 × 10^5^ per sample) were harvested, centrifuged, and incubated with MTG at a final concentration of 120 nM in pre-warmed growth medium for 30 min at 37 °C in 5% CO_2_, in the dark, according to the manufacturer’s protocol. Following staining, cells were washed twice with growth medium, resuspended in FACS buffer, and immediately analyzed by flow cytometry. Flow cytometry was performed on a Northern Lights cytometer (Cytek Biosciences, Fremont, CA, USA), and data were analyzed using FlowJo software 10.10.0 (BD Biosciences, San Jose, CA, USA). At least 10,000 events were collected per sample. Mitochondrial content was quantified as the geometric mean fluorescence intensity (gMFI) of MTG in live cell populations after exclusion of debris and doublets.

### 2.8. Statistical Analysis

Analyses were conducted using GraphPad Prism 10 software. Data were first tested for normality using the Shapiro–Wilk test. For datasets that did not meet the assumption of a normal distribution, comparisons between groups were performed using the non-parametric Mann–Whitney U test. Statistical significance was defined as *p* < 0.05.

## 3. Results

### 3.1. Patient Cohort

We studied fibroblasts from six genetically confirmed DLD-deficient patients carrying distinct pathogenic variants (Figure 1, Table 1, and Supplementary Figure S1), five of whom were male and one female. The cohort illustrates the broad clinical heterogeneity of the disorder, ranging from severe neonatal presentations with early-onset metabolic crises and profound developmental delay, to milder phenotypes, and even unexpectedly severe disease in a patient harboring the common c.685G>T; p.G229C variant in homozygosity, typically associated with a mild course [3,16].

Patient 1 (Pt1 homozygous for c.1436A>T; p.D479V), a Bedouin male, was born to consanguineous parents (first cousins), and had a skin biopsy performed at 5 years of age. He presented with severe neonatal lactic acidosis and hypoglycemia at 24 h of life, followed by recurrent metabolic decompensations, seizures, spasticity, kidney stones, graft versus host disease, and profound developmental delay, currently with no eye contact. He attained minimal developmental milestones and is treated with a modified ketogenic diet, sodium bicarbonate, levetiracetam, clonazepam, vitamin D, and potassium citrate.

Patient 2 (Pt2, homozygous for c.1436A>T; p.D479V), a Bedouin female, was born to consanguineous parents (second cousins) with a family history of perinatal losses due to DLD deficiency, and had a skin biopsy performed at 4 years of age. She presented with severe lactic acidosis on the first day of life. She exhibits severe disease with recurrent seizures, generalized spasticity with dystonia, microcephaly, failure to thrive, and profound developmental delay; At 7 years of age she is unable to sit or walk independently. She is treated with a modified ketogenic diet, sodium bicarbonate, levetiracetam, vitamin D, IV bisphosphonates, and potassium citrate.

Patient 3 (Pt3, homozygous for c.1123G>A; p.E375K), an Ashkenazi Jewish male, is the second affected sibling in the family, and had a skin biopsy performed at 3 months of age. He experienced multiple hospitalizations for recurrent metabolic crises in early childhood, triggered by vomiting, anorexia, and infections. With supportive therapy (carnitine, sodium bicarbonate, vitamin B1, vitamin D, muscle relaxants, and potassium citrate), he attained near-age-appropriate developmental milestones, attends school, and experienced a significant reduction in crisis frequency. Presently, his main complaints are fatigue and weakness with minimal activity.

Patient 4 (Pt4, compound heterozygous for c.105insA; p.Y35X/c.685G>T; p.G229C), an Ashkenazi Jewish male, displays a milder phenotype, with early childhood hospitalizations for vomiting, elevated liver enzymes, developmental delay, and hypotonia, and had a skin biopsy performed at 20 years of age. He has remained stable for over a decade without metabolic decompensation and is managed with riboflavin and B-complex supplementation.

Patient 5 (Pt5, compound heterozygous for c.158G>A; p.G53E/c.685G>T; p.G229C), a mixed Ashkenazi Sephardic Jewish male, presented with recurrent metabolic decompensations characterized by vomiting, altered mental status, lactic acidosis, hyperammonemia, and elevated liver enzymes. He had a skin biopsy performed at 5 years of age. He later suffered a severe crisis resulting in involuntary eye movements and dysarthria, which was followed by gradual recovery. He remains with residual dysarthria and incoordination, infrequently exacerbated with abnormal eye and neck movement. His current therapy includes riboflavin, tetrabenazine, and risperidone (for hyperactivity).

Patient 6 (Pt6, homozygous for c.685G>T; p.G229C), a mixed Ashkenazi, Northern African Jewish male, had a skin biopsy performed at 2.5 years of age. Despite carrying the common Ashkenazi Jewish variant typically associated with a milder course, he presents with severe disease. His first decompensation occurred at 3 days of life with hypoglycemia and focal seizures. Clinical features include recurrent infection-triggered decompensations; however, he developed normally. At the age of two years, the patient experienced a metabolic crisis triggered by fever. During this episode the patient had severe hypoglycemia, status epilepticus, encephalopathy, and hyperammonemia. He required prolonged admission at PICU. This episode resulted in brain damage, and the patient remained with significant persistent neurological impairment. At 3.5 years he is able to walk independently, though somewhat clumsily, he is able to speak sentences of 2–3 words, and he continues to make developmental progress, although he remains with moderate cognitive delay. Treatment includes a ketogenic diet, carnitine, riboflavin, antiepileptics, and vitamin B6.

### 3.2. Mitochondrial Respiration in Patient-Derived Fibroblasts

Fibroblast cell lines were established from skin biopsies of patients 1–6 with distinct mutations and clinical phenotypes (Figure 1 and Supplementary Figure S1). High-resolution respirometry was applied to evaluate oxygen consumption under defined substrate–inhibitor conditions (Supplementary Figures S2 and S3). At routine respiration, Pt6 exhibited a significant reduction relative to control, while Pt1 and Pt2 (the most clinically severe) showed a trend toward lower rates (*p* = 0.063; Figure 2A). Under maximal respiratory capacity, all six patient-derived lines demonstrated significantly reduced oxygen consumption compared with controls (Figure 2B). ATP synthase-linked respiration (oligomycin) did not differ significantly between patient and control fibroblasts (Supplementary Figure S4), consistent with a primary defect in matrix dehydrogenase activity rather than ATP synthase function.

When complex-specific activities were analyzed, Pt1 and Pt2 showed marked reductions in both complex I- and complex II-linked respiration (Figure 2C,D). However, as expected, all six patient lines exhibited a uniformly diminished response to complex I inhibition relative to controls (Figure 2E). This pattern reflected similar trends in the complex I/II respiration ratio: patients 1–4 showed a significant shift toward reduced complex I contribution, Pt5 displayed a non-significant trend (*p* = 0.073), and Pt6 did not differ from control (Figure 2F). Given that the DLD E3 subunit is integral to PDHc and αKGDHc, generating NADH that feeds electrons into complex I, these findings indicate that impaired E3 function limits electron supply to complex I, producing the consistent bioenergetic defect observed. Collectively, mitochondrial respiration is globally impaired in DLD-deficient fibroblasts, with maximal respiration and complex I function most prominently affected, and the greatest defects observed in the most clinically severe patients.

### 3.3. Distinguishing PDHc- and αKGDHc-Linked Respiration

To further dissect the contribution of individual mitochondrial dehydrogenase complexes (PDHc and αKGDHc) to the overall respiratory defect in DLD deficiency, we compared our initial respiratory measurements (Figure 2) with DLD enzymatic activity (Supplementary Table S1). These measurements correlated with respiration seen in patient fibroblasts. However, the DLD enzymatic assay and the common approach respiratory measurement (Figure 2) did not allow us to distinguish the specific contribution of PDHc and αKGDHc activities. Therefore, we developed a substrate–inhibitor sequence designed to differentiate between PDHc- and αKGDHc-linked respiration within the same experimental setup. In this protocol, the addition of pyruvate and ADP was used to specifically evaluate PDHc-dependent respiration, while subsequent addition of glutamate enabled assessment of αKGDHc-linked activity (Figure 3A and Supplementary Figures S5 and S6). Importantly, αKGDHc-linked respiration was quantified immediately after the addition of glutamate; malate was added only afterward to sustain downstream TCA cycle turnover. Therefore, the increase in respiration observed after glutamate and subsequent malate addition reflects combined NADH-linked flux from both αKGDHc and malate dehydrogenase; accordingly, higher values in some patient lines with glutamate only, could represent a compensatory maximal αKGDHc activity. Notably upon malate addition, oxygen consumption did not increase as dramatically as in the control sample, which may reflect them already having reached their αKGDHc maximal capacity (Supplementary Figure S6). Using this approach, we found that patients 1–4 exhibited significantly reduced respiration following pyruvate and ADP addition, indicating impaired PDHc-dependent flux (Figure 3B). In contrast, only the two most clinically severe patients (Pt1 and Pt2) demonstrated a marked reduction in respiration after the addition of glutamate, reflecting impaired αKGDHc activity in addition to PDHc dysfunction (Figure 3C). The combined deficit in both PDHc- and αKGDHc-linked respiration in Pt1 and Pt2 likely contributes to their more severe clinical presentation, whereas the other patients displayed isolated PDHc impairment without significant αKGDHc involvement.

### 3.4. Mitochondrial Mass Assessment

To determine whether the reduced respiratory capacity observed in DLD-deficient fibroblasts could be attributed to differences in mitochondrial abundance rather than intrinsic functional defects, mitochondrial content was assessed using two complementary approaches. First, mtDNA copy number was quantified by qPCR. While most patient-derived fibroblast lines showed comparable mtDNA levels to controls, Pt5 exhibited a significantly reduced mtDNA copy number, and Pt2 displayed a near-significant trend (*p* = 0.051) toward lower mtDNA content (Supplementary Figure S7). Second, mitochondrial mass was measured by flow cytometry using MTG, which revealed a similar pattern between patient and control fibroblasts (Supplementary Figure S8). Together, these findings indicate that the respiratory defects observed in DLD-deficient cells are not due to global reductions in mitochondrial mass, but rather reflect intrinsic mitochondrial functional impairments.

## 4. Discussion

DLD deficiency is a rare and clinically heterogeneous mitochondrial disorder, with phenotypes ranging from severe neonatal metabolic crises to more stable hepatic or neurological courses [16,17,18]. This variability has historically complicated genotype–phenotype correlations, in part because DLD serves as the shared E3 subunit of multiple mitochondrial enzyme complexes, such as the pyruvate dehydrogenase complex (PDHc), α-ketoglutarate dehydrogenase complex (αKGDHc), branched-chain α-keto acid dehydrogenase complex (BCKDHc), and glycine cleavage system [5,19]. In this study, we applied high-resolution respirometry to patient-derived fibroblasts to comprehensively profile mitochondrial respiration and, critically, to distinguish the relative contributions of PDHc- and αKGDHc-linked flux to bioenergetic impairment. Previous studies in DLD deficiency have primarily relied on enzymatic activity assays or biochemical measurements performed in isolation [3,9], without resolving whether respiratory defects stem from impaired PDHc or αKGDHc activity. This limitation has hindered important mechanistic insight into the disease. Our data demonstrate that maximal respiratory capacity and complex I-linked respiration are consistently reduced across all patient-derived lines, regardless of genotype. Moreover, all patients exhibited a uniformly blunted response to complex I inhibition, supporting the concept that DLD dysfunction restricts electron input into complex I, thereby creating a shared bioenergetic signature in DLD deficiency. This finding aligns with the central role of DLD in generating NADH through PDHc and αKGDHc activity, which directly fuels complex I of the mitochondrial respiratory chain [10]. Although reduced NADH supply from impaired PDHc and αKGDHc activity provides a direct explanation for the diminished complex I-linked respiration and blunted rotenone response observed across all patient lines, we also acknowledge the possibility of a secondary complex I dysfunction. Recent studies on other OXPHOS disorders have demonstrated that severe mitochondrial dysfunction can destabilize complex I, and similar secondary changes have been reported in disorders of fatty-acid oxidation [20,21] and TCA-cycle enzyme deficiencies [22,23]. In addition, defects in mitochondrial protein synthesis have been shown to downregulate mitochondrial ribosomal proteins and further impair complex I assembly [24,25]. A key advance of this work is the application of a modified SUIT protocol to resolve PDHc- from αKGDHc-linked respiration within the same experimental setup. This analysis revealed that impaired PDHc activity was present in most patients, whereas αKGDHc dysfunction was restricted to the most clinically severe cases. The differences observed between PDHc- and αKGDHc-linked respiration in our fibroblast model likely reflect differences in metabolic flux and the amount of residual DLD activity or protein abundance. This distinction provides a mechanistic explanation for the variability in disease severity: the combined impairment of both PDHc and αKGDHc may underlie the profound neurodegeneration and metabolic instability observed in patients with dimer interface mutations such as c.1436A>T; p.D479V, while isolated PDHc dysfunction may account for milder phenotypes. Importantly, the involvement of αKGDHc in the most severe patients also offers a plausible explanation for their poor response to ketogenic diet therapy (Table 1), as ketone metabolism still requires intact αKGDHc function to support oxidative metabolism and maintain neuronal energy homeostasis. These findings are consistent with structural predictions that dimer interface variants destabilize E3 function more broadly, whereas other variants (e.g., c.685G>T; p.G229C) exert more restricted effects [3,16,26].

Interestingly, Pt6, who is homozygous for the common Ashkenazi Jewish founder mutation c.685G>T; p.G229C, typically associated with a milder, predominantly hepatic phenotype, presented with unexpectedly severe clinical features. In line with this, Pt6 was the only patient to show a significant reduction in both routine and maximal respiration compared to controls, suggesting a broader bioenergetic defect. The phenotypic differences between patients carrying the c.685G>T; p.G229C variant (Pt4–Pt6) probably reflect differences in total residual DLD activity. Consistent with this, our direct DLD enzymatic assays (Supplementary Table S1), demonstrated that control fibroblasts displayed activity within the normal range (75.29 mU/mg), whereas Pt4, who is compound heterozygous for c.685G>T; p.G229C and a premature stop mutation (c.105insA; p.Y35X), showed markedly reduced activity (2.46 mU/mg), and Pt6 retained substantially higher residual activity (10.99 mU/mg). These quantitative differences align with the graded respiratory phenotypes observed in Figure 2 and support the interpretation that residual DLD activity contributes to clinical variability. The atypical phenotype of Pt6 raises the possibility that additional genetic, epigenetic, or environmental modifiers may exacerbate mitochondrial dysfunction in this patient, resulting in a more severe disease course than generally observed for this variant.

This observation is consistent with previous reports showing that patients harboring the same c.685G>T; p.G229C variant can present with strikingly variable phenotypes, ranging from asymptomatic carriers to fulminant hepatic failure requiring urgent liver transplantation in five affected family members [26]. Such variability underscores the complexity of genotype–phenotype correlations in DLD deficiency and suggests that disease severity cannot be predicted solely by genotype. Instead, secondary modifiers and environmental stressors may critically influence the clinical trajectory. Beyond the mitochondrial defects assessed in this study, it is also important to recognize that DLD participates in additional enzyme systems, including BCKDHc and the glycine cleavage system, and dysfunction in these pathways [18] may further modulate disease expression. Moreover, a nuclear-localized pool of αKGDHc has been shown to regulate histone lysine succinylation and transcriptional programs [27,28], providing an additional layer of metabolic–epigenetic regulation that may contribute to phenotypic variability in DLD deficiency. Although these pathways were not directly assessed here, they likely interact with the mitochondrial bioenergetic defects we observed to shape the overall clinical presentation. Together, these findings highlight that while c.685G>T; p.G229C is generally linked to a mild phenotype, it can manifest as severe multisystem disease under certain conditions. Recognizing this variability is essential for patient management and suggests that a more personalized approach, taking into account potential modifying factors, will be required to improve prognostic accuracy and therapeutic strategies in DLD deficiency.

Our data indicate that the respiratory defects observed in DLD-deficient fibroblasts are not primarily attributable to reduced mitochondrial mass. While mitochondrial content assessed by MTG staining showed no significant differences between patients and the control, mtDNA copy number analysis revealed a mild reduction in certain patient lines, most notably in Pt5, with a trend toward lower values in Pt2 (*p* = 0.051). This partial reduction in mtDNA copy number without a corresponding decrease in mitochondrial mass may reflect compensatory mitochondrial biogenesis or structural maintenance despite impaired replication or mtDNA stability. Similar uncoupling between mtDNA content and mitochondrial volume has been reported under conditions of oxidative stress and defective mtDNA replication [29]. Alternatively, altered mitochondrial turnover or selective mitophagy may contribute to this discrepancy [30]. These findings underscore the importance of assessing mitochondrial abundance using multiple complementary approaches, combining mtDNA quantification with organelle mass, to accurately interpret bioenergetic phenotypes in mitochondrial disorders. Collectively, our results support that the respiratory impairments in DLD-deficient fibroblasts are driven by intrinsic enzymatic dysfunction rather than a global reduction in mitochondrial content.

Clinically, these findings highlight the importance of considering enzyme-specific contributions to pathophysiology when evaluating patients with DLD deficiency. Functional stratification of PDHc- versus αKGDHc-linked defects could inform prognosis and therapeutic strategies. For instance, patients with isolated PDHc impairment may respond differently to dietary or pharmacologic interventions compared to those with combined PDHc/αKGDHc dysfunction (Table 1). Furthermore, the consistent complex I defect across the cohort suggests that therapies targeting NADH reoxidation or complex I bypass strategies may hold promise for DLD deficiency and for other PDHc and αKGDHc defects, similar to approaches under exploration for other primary mitochondrial diseases [31,32].

This study has several limitations that should be acknowledged. First, the cohort size was relatively small, reflecting the rarity of DLD deficiency, and therefore, the generalizability of our findings requires validation in larger patient populations. Second, while fibroblasts provide an accessible model to assess mitochondrial function, they may not fully capture the tissue-specific pathophysiology observed in the brain, liver, and muscle of affected patients. Third, our assays focused primarily on bioenergetic parameters, and additional layers of regulation such as redox balance, post-translational modifications, and metabolic signaling networks were not directly addressed [11,33]. Future studies integrating multi-omics approaches, including proteomics, metabolomics, and transcriptomics, will be essential to delineate the broader consequences of DLD dysfunction [11,34]. In addition, patient-derived neuronal and hepatic models, induced pluripotent stem cell (iPSC)-based systems, or organoids could provide more relevant platforms to investigate tissue-specific effects [35,36,37]. Finally, expanding functional profiling to include therapeutic interventions, such as metabolic supplementation or pharmacological modulators, may yield mechanistic insights into differential treatment responses and inform the development of personalized therapies.

In summary, we show that DLD deficiency produces a reproducible bioenergetic signature characterized by impaired maximal respiration and reduced complex I-linked flux, independent of mitochondrial mass. By developing an integrated assay to distinguish PDHc- and αKGDHc-linked respiration, we provide mechanistic insight into how specific variants differentially affect enzyme function and clinical outcomes. This framework not only advances understanding of DLD deficiency but also establishes a platform for evaluating therapeutic interventions in patient-derived cells.

## Figures and Tables

**Table 1:** Patients’ phenotypic characterization. Pt—Patient; P—protein; P/w—presented with; MRI—magnetic resonance imaging; EEG—electroencephalogram; DQ—developmental quotient. Ketogenic diet highlighted in underline.

Pt	Gender and Ethnicity	*DLD* Genotype (P)	Onset	Clinical Presentation	MRI	EEG	Development	Treatment	Outcomes
Pt1	M, Bedouin	D479V/ D479V	1 day	P/w severe lactic acidosis. Later developed recurrent crises of lactic acidosis and hypoglycemia (mostly triggered by infections). Developed seizures, spasticity, and failure to thrive.	N/A	Hypsarrhythmia	Severe global developmental delay with minimal attained milestones.	Sodium bicarbonate, anti-epileptics (Keppra, clonazepam), vitamin D, potassium citrate. Modified ketogenic diet (1:0.82).	Normalization of EEG.
Pt2	F, Bedouin	D479V/ D479V	At birth	P/w severe lactic acidosis (pH 6.9). Later developed recurrent crises of lactic acidosis (mostly triggered by infections). Developed seizures, spasticity, failure to thrive and osteopenia.	Basal nuclei hyperintensity, cerebral ventriculomegaly.	Epileptiformic discharges (right temporal and frontal).	Severe global developmental delay with minimal attained milestones.	Sodium bicarbonate, anti-epileptics (Keppra), vitamin D, bisphosphonates (IV), and potassium citrate. Modified ketogenic diet (1:0.76).	Normalization of EEG.
Pt3	M, Ashkenazi Jewish	E375K/ E375K	3 months	P/w lactic acidosis and elevated liver enzymes, followed by intermittent crises until age 5 with lactic acidosis, hypoglycemia, vomiting and elevated liver enzymes.	N/A	N/A	Primarily motor developmental delay. Mild dysarthria and dystonia.	Carnitine, sodium bicarbonate, Thiamine, vitamin D, muscle relaxants, and potassium citrate. Modified ketogenic diet (1:0.6).	Crises free > 7 years.
Pt4	M, Ashkenazi Jewish	Y35X/ G229C	1 year	Recurrent crises of lactic acidosis, hypoglycemia, vomiting, elevated liver enzymes, hyperammonemia.	N/A	N/A	Motor developmental delay, hypotonia, scoliosis.	Riboflavin and Vitamin complex B. Received DCA which was discontinued due to concerns of neuropathy.	Crisis free > 10 years.
Pt5	M, Mixed AshkenaziSephardic Jewish	G53E/ G229C	12 h	P/w lactic acidosis, hypoglycemia and hypotonia, followed by intermittent crises of lactic acidosis, hypoglycemia, vomiting, elevated liver enzymes, hyperammonemia, and hypotonia.	Gliotic-cystic changes bilaterally in the basal ganglia, diffusion restriction bilateral foci in the globus pallidus.	Normal	Speech and motor delays, limb dystonia.	Riboflavin, tetrabenazine, risperidone. Diet: minimize carbohydrates from table food.	Improved appetite and weight gain. Improvement of focal lesions (MRI). Multiple admission for crises involving confusion and worsening of movement disorders.
Pt6	M, Mixed Ashkenazi, Northern African Jewish	G229C/ G229C	3 days	P/w severe hypoglycemia and seizures associated with hyperammonemia. Recurrent crises of hypoglycemia, seizures, vomiting, elevated liver enzymes, hyperammonemia and encephalopathy.	Global brain atrophy.	Multifocal epileptic activity with generalized slowing.	Global developmental delay. Intellectual disability (DQ 40).	Carnitine, riboflavin, anti-epileptics and vitamin B6. Full ketogenic diet.	No metabolic crises, and persistent developmental improvement.

## Data Availability

The original contributions presented in this study are included in the article/Supplementary Material. Further inquiries can be directed to the corresponding author.

## Supplementary Materials

The following supporting information can be downloaded at: https://www.mdpi.com/article/10.3390/antiox15010019/s1.

## Abbreviations

The following abbreviations are used in this manuscript

DLD	Dihydrolipoamide dehydrogenase
PDHc	Pyruvate dehydrogenase complex
αKGDHc	α-ketoglutarate dehydrogenase complex
BCKDHc	Branched-chain α-keto acid dehydrogenase complex
mtDNA	Mitochondrial DNA
FAD	Flavin adenine dinucleotide
NAD	Nicotinamide adenine dinucleotide
ATP	Adenosine triphosphate
DMEM	Dulbecco’s Modified Eagle’s Medium
FBS	Fetal bovine serum
SUIT	Substrate–uncoupler–inhibitor titration
OXPHOS	Oxidative phosphorylation
ADP	Adenosine diphosphate
FCCP	Carbonyl cyanide p-trifluoromethoxyphenylhydrazone
ETS	Electron transport system
nDNA	Nuclear DNA
CT	Cycle threshold
MTG	MitoTracker Green
FACS	Fluorescence-activated cell sorting
gMFI	Geometric mean fluorescence intensity
qPCR	Quantitative polymerase chain reaction
MTS	Mitochondrial targeting sequence
Ctrl	Control
PICU	Pediatric Intensive Care Unit
P	Protein
Pt	Patient
P/w	Presented with
MRI	Magnetic resonance imaging
EEG	Electroencephalogram
DQ	Developmental quotient
iPSC	Induced pluripotent stem cell
