# Supplementary material for: Bioenergetic Signatures of DLD Deficiency: Dissecting PDHc- and α-KGDHc-Linked Defects
Source: Antioxidants (Basel). 2025 Dec 22;15(1):19. doi: 10.3390/antiox15010019 (PMC12837974; doi:10.3390/antiox15010019)
Supplement: Supplementary file 1 [file antioxidants-15-00019-s001.zip › antioxidants-3973859-supplementary.pdf]

antioxidants

Supplementary Materials for:

Bioenergetic Signatures of DLD Deficiency: Dissecting PDHc and α-KGDHc Linked Defects

Yarden Haham Zarbib [1,2,†], Shira Huri Ohev-Shalom [1,†], Shani Kassia Lyskov [1,2], Yuval Mazor [1,2], Mika Anekstein-Spigel [1,2], Nechama Shalva [3], Ronen Spiegel [4,5], Orna Staretz-Chacham [6], Joshua Manor [2,3], Ann Saada [7,8], Rachel Rock [3], Yair Anikster [2,3] and Tal Yardeni [1,*]

[1] Metabolic Center, Sheba Medical Center, Tel-Hashomer, Ramat Gan 52621, Israel; yarden.haham@sheba.health.gov.il (Y.H.Z.); shira.huriohevshalom@sheba.health.gov.il (S.H.O.-S.); shanilyskov@mail.tau.ac.il (S.K.L.); yuval.mazor@sheba.health.gov.il (Y.M.); mika.anekstein-spigel@sheba.health.gov.il (M.A.-S.)
[2] Faculty of Medicine and Life Sciences, Tel Aviv University, Tel Aviv 69978, Israel; yehoshua.manor@sheba.health.gov.il (J.M.); yair.anikster@sheba.health.gov.il (Y.A.)
[3] Metabolic Disease Unit, Edmond and Lily Safra Children's Hospital, Sheba Medical Center, Ramat Gan 52621, Israel; nechama.shalva@sheba.health.gov.il (N.S.); rachel.rock@sheba.health.gov.il (R.R.)
[4] Department of Pediatrics B, Emek Medical Center, Afula 23100, Israel; spiegel_ro@clalit.org.il
[5] Rappaport Faculty of Medicine, Technion, Haifa 3525433, Israel
[6] Metabolic Clinic, Pediatric Division, Soroka University Medical Center, Ben Gurion University, Beer Sheva 84105, Israel; staretz@bgu.ac.il
[7] Department of Genetics, Hadassah Medical Center and Faculty of Medicine, Hebrew University of Jerusalem, Jerusalem 91220, Israel; ann.saadareisch@mail.huji.ac.il
[8] Department of Medical Laboratory Sciences, Jerusalem Multidisciplinary College, Jerusalem 9422408, Israel
* Correspondence: tal.yardeni@sheba.health.gov.il; Tel.: +972-3-5302371
† These authors contributed equally to this work.

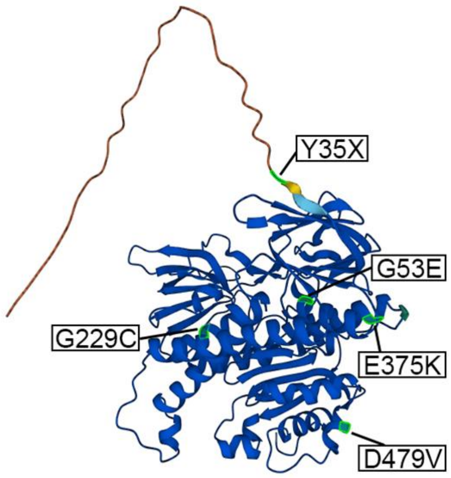

Supplementary Figure S1. Structural mapping of patient derived DLD variants on the predicted human DLD protein model. Shown is the AlphaFold predicted three dimensional (3D) structure of human DLD (UniProt P09622). Patient specific variants identified in this study (c.105insA; p.Y35X, c.158G>A; p.G53E, c.685G>T; p.G229C, c.1123G>A; p.E375K, c.1436A>T; p.D479V) are highlighted in green on the structural model. The N-terminal mitochondrial targeting sequence, predicted to be unstructured, is shown in brown. Structural coordinates were obtained from the AlphaFold Protein Structure Database (https://alphafold.ebi.ac.uk/entry/AF-P09622-F1).

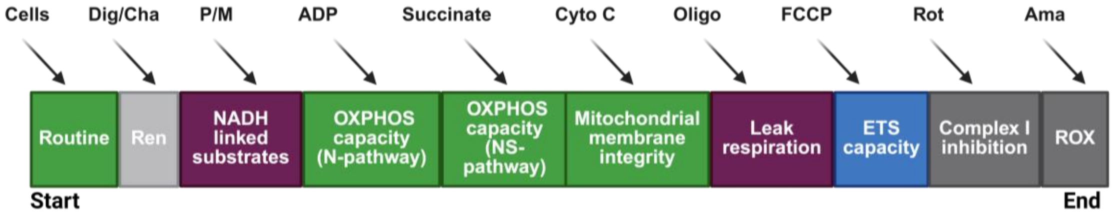

Supplementary Figure S2. High-resolution respirometry substrate–uncoupler–inhibitor titration (SUIT) protocol used to assess mitochondrial function in permeabilized fibroblasts. Schematic overview of the sequential substrate and inhibitor additions used to quantify distinct respiratory states. Routine respiration was recorded in intact cells prior to permeabilization. Digitonin or α-chaconine (Dig/Cha) was then added to selectively permeabilize the plasma membrane, after which Ren was measured, representing residual endogenous oxygen consumption prior to substrate addition. Pyruvate (P) and malate (M) initiated NADH-linked respiration (N-pathway substrates), followed by ADP to stimulate OXPHOS capacity (N-pathway). Succinate addition enabled assessment of OXPHOS capacity (NS-pathway) through convergent electron flow via complexes I and II. Cytochrome c (Cyto C) was used to verify mitochondrial outer membrane integrity. Oligomycin (Oligo) inhibited ATP synthase to measure LEAK respiration. FCCP titrations were used to determine maximal electron transfer system (ETS) capacity. Rotenone (Rot) inhibited complex I, and antimycin A (Ama) inhibited complex III to define residual oxygen consumption (ROX). Created in BioRender. Yardeni, T. (2025) *https://BioRender.com/4u89mh2*.

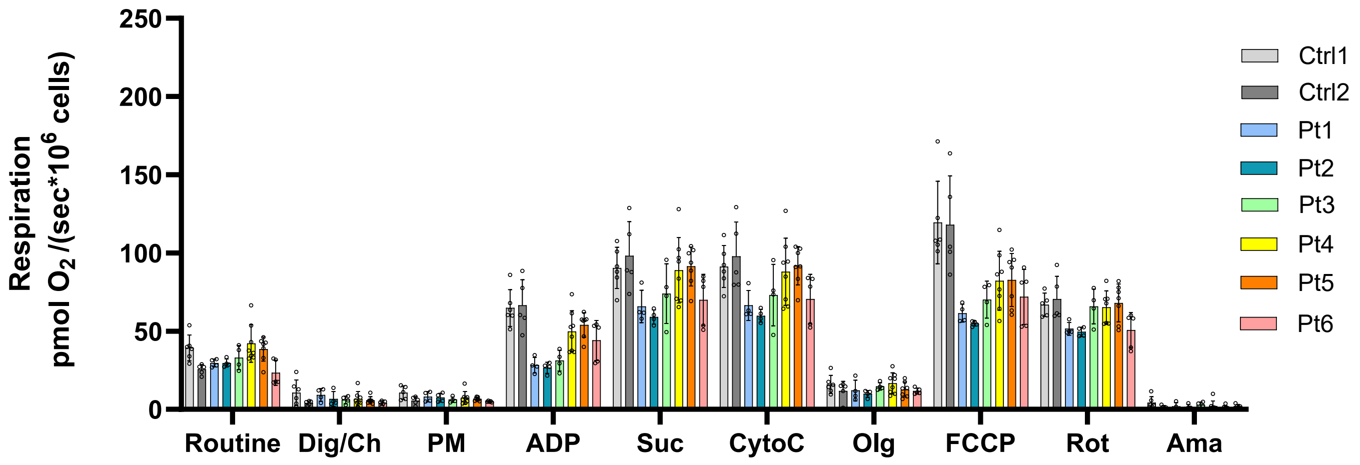

Supplementary Figure S3. Comprehensive analysis of mitochondrial respiration in control and patient-derived fibroblasts: Oxygen consumption rates were measured using high-resolution respirometry under sequential substrate–inhibitor additions. Routine, basal respiration; Dig/Ch, cell permeabilization (digitonin and α-chaconine); PM, complex I–linked leak respiration supported by pyruvate and malate; ADP, ADP-stimulated OXPHOS capacity; Suc, succinate-supported OXPHOS (complex II activity); CytoC, addition of cytochrome c to assess mitochondrial outer membrane integrity; Olg, oligomycin-induced leak respiration; FCCP, maximal uncoupled electron transport capacity; Rot, rotenone-mediated complex I inhibition demonstrating complex II–linked respiration; Ama, antimycin A inhibition of complex III indicating residual non-mitochondrial oxygen consumption. Each open circle represents an independent experiment (N=4-8 repeats per sample). Control fibroblasts are denoted as Ctrl1 and Ctrl 2; patient-derived lines are indicated as Pt1–Pt6. Data are normalized to cell number. Negative oxygen flux values in the Ama stage were set to zero.

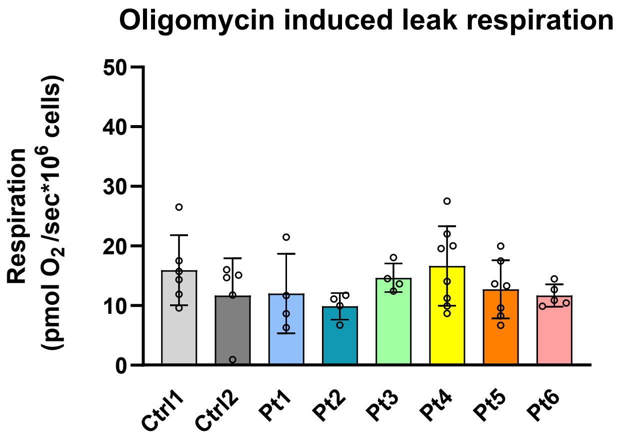

Supplementary Figure S4: Oligomycin-induced LEAK respiration in fibroblasts from patients with DLD deficiency. ATP synthase independent (LEAK) respiration was quantified in controls (Ctrl1 and Ctrl2) and patient fibroblasts (Pt1–Pt6) following inhibition of ATP synthase with oligomycin. Respiration was measured using high-resolution respirometry (Oroboros O2k). Each open circle represents an independent experimental run (N=4-8). All data were normalized to cell number. No statistically significant differences were observed between patient lines and controls (Mann–Whitney U test).

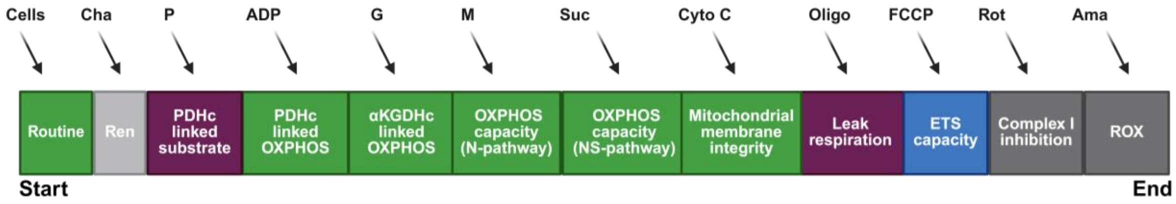

Supplementary Figure S5: Schematic overview of the substrate uncoupler inhibitor titration (SUIT) protocol used to dissect PDHc and αKGDHc linked respiration in permeabilized fibroblasts. The sequence of additions used for high resolution respirometry is shown from left to right. Routine respiration was first recorded in intact fibroblasts. α-chaconine (Cha) was then added to selectively permeabilize the plasma membrane, after which Ren was measured, representing residual endogenous oxygen consumption prior to substrate addition. Pyruvate (P) served as the PDHc-linked substrate, followed by ADP to stimulate PDHc-linked OXPHOS. Glutamate (G) was added to generate α-ketoglutarate via aminotransferase activity, enabling quantification of αKGDHc linked OXPHOS. Malate (M) was introduced to sustain downstream NADH linked flux, followed by succinate (Suc) to evaluate NS-pathway OXPHOS via convergent electron input through complexes I and II. Cytochrome c (Cyto C) addition verified mitochondrial outer membrane integrity. Oligomycin (Oligo) inhibited ATP synthase to measure LEAK respiration, and FCCP was titrated to determine maximal ETS capacity. Finally, rotenone (Rot) and antimycin A (Ama) were added to quantify complex I inhibited respiration and residual oxygen consumption (ROX), respectively. Created in BioRender. Yardeni, T. (2025) https://BioRender.com/milazm5.

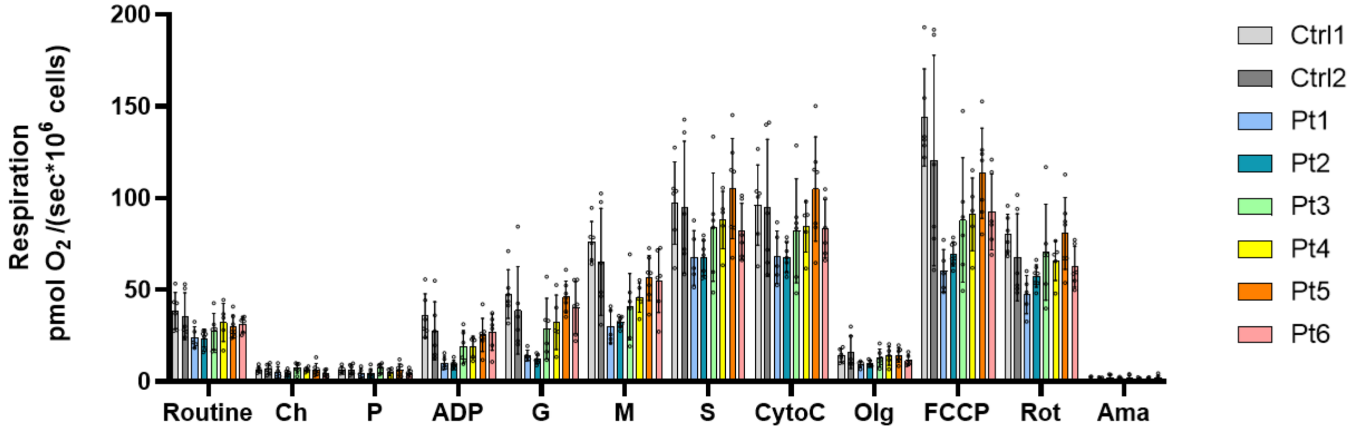

Supplementary Figure S6. Distinguishing PDHc and αKGDHc linked mitochondrial respiration in control and DLD-deficient fibroblasts: High-resolution respirometry was performed using a modified SUIT protocol designed to separately assess PDHc and αKGDHc linked respiration. Sequential substrate and inhibitor additions were performed as follows: Routine, basal respiration; Ch, cell permeabilization with α-chaconine; P, pyruvate-driven complex I-linked leak respiration; ADP, ADP-stimulated respiration reflecting PDHc activity; G, glutamate-supported respiration representing αKGDHc activity; M, malate addition sustaining total complex I-linked flux; S, succinate-supported respiration assessing complex II-linked OXPHOS capacity; CytoC, cytochrome c addition to verify mitochondrial outer membrane integrity; Olg, oligomycin-induced leak respiration; FCCP, maximal uncoupled electron transport capacity; Rot, rotenone inhibition of complex I indicating complex II-linked respiration; Ama, antimycin A inhibition of complex III indicating residual non-mitochondrial oxygen consumption. Each open circle represents an independent experimental run (N=5-7 repeats per sample). Control fibroblasts are denoted as Ctrl1-Ctrl2; patient-derived lines as Pt1–Pt6. Data are normalized to cell number. Negative oxygen flux values in the Ama stage were set to zero.

Sample	DLD activity (mU/mg)
**Ctrl**	**75.29**
**Pt1**	**0.53**
**Pt2**	**1.02**
**Pt3**	**2.54**
**Pt4**	**2.4**
**Pt5**	**5.45**
**Pt6**	**10.99**

Supplementary Table S1: DLD activity was measured in fibroblast lysates from same day control and from Pt1–Pt6 patients. DLD activity was measured in fibroblast lysates from same-day controls and from Pt1–Pt6 DLD-deficiency patients. Control fibroblasts showed DLD activity within the expected normal range (75.90 ± 19.69 mU/mg)[1], whereas all patient samples exhibited markedly reduced activity. Enzymatic activity is expressed as mU/mg total protein, with each sample measured in 2–3 technical replicates (N=2–3).

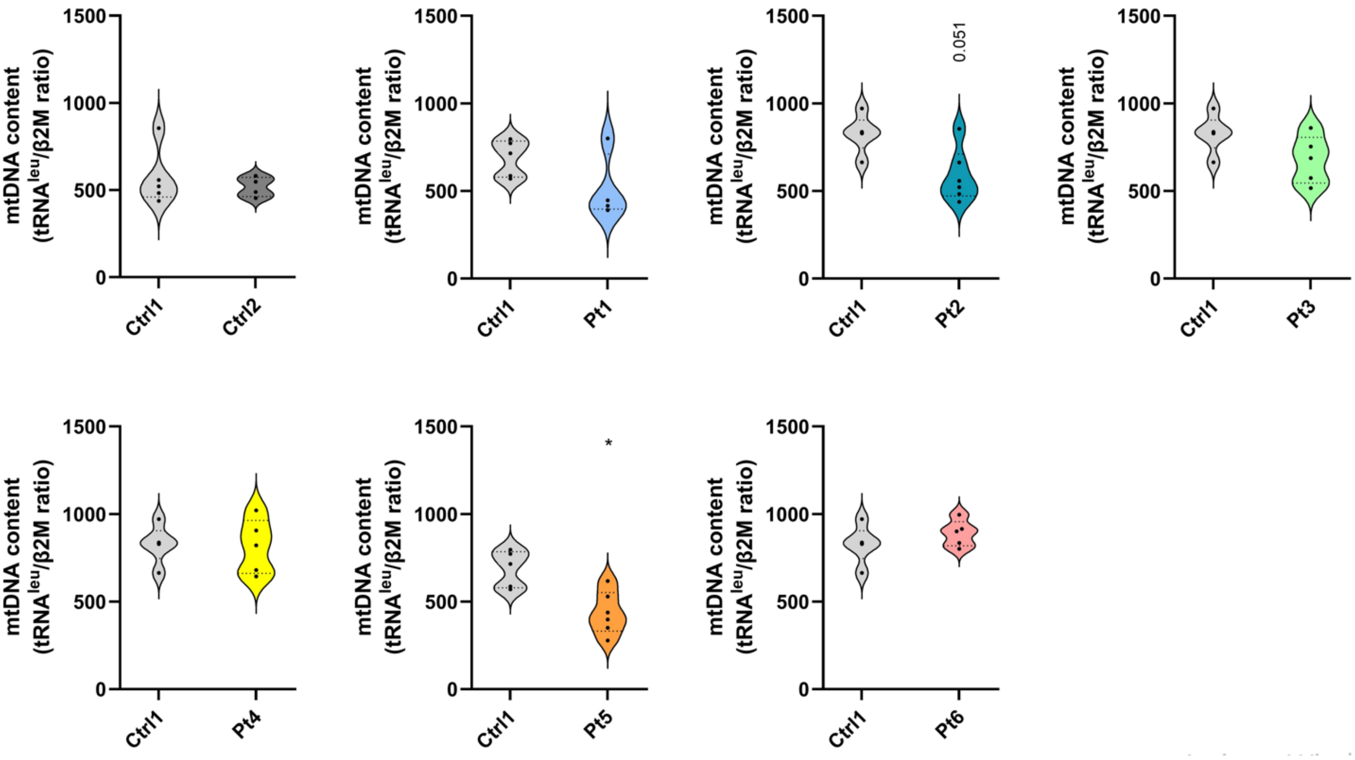

Supplementary Figure S7. Mitochondrial DNA (mtDNA) copy number in fibroblasts from DLD-deficient patients: (A-F) Relative mtDNA content was quantified by qPCR and expressed as the ratio of mitochondrial *tRNA*^{Leu} to nuclear *β2M* (β2-microglobulin) for each patient-derived fibroblast line (Pt1–Pt6), and Ctrl 2 compared with control (Ctrl 1). Each dot represents an independent biological replicate (N=4-6 repeats per sample). Data are shown as violin plots indicating distribution, median, and interquartile range. Statistical significance was determined using the Mann–Whitney U test. P < 0.05 was considered significant and numerical P values (0.05 < P < 0.1) are indicated on the plots.

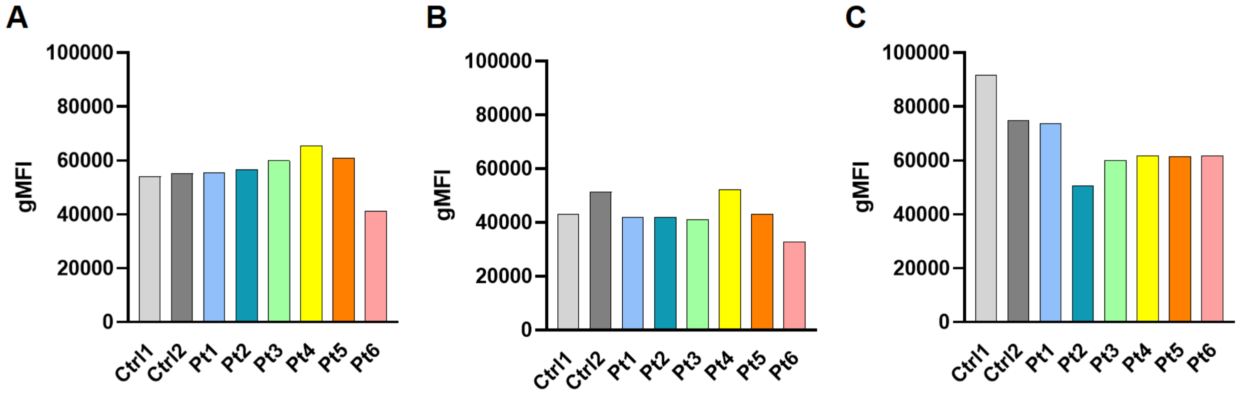

Supplementary Figure S8. Flow cytometric analysis of mitochondrial content using MitoTracker Green (MTG): Mitochondrial mass was assessed in control and DLD-deficient fibroblasts using MTG, a potential independent fluorescent probe. The Y-axis represents the geometric mean fluorescence intensity (gMFI) of MTG staining. Data are representative of three independent experiments (A-C)**,** each showing comparable gMFI values across all fibroblast lines, indicating that mitochondrial content is not altered in DLD-deficient cells.

Reference:

1. Saada, A.; Aptowitzer, I.; Link, G.; Elpeleg, O.N. ATP Synthesis in Lipoamide Dehydrogenase Deficiency. *Biochem Biophys Res Commun* **2000**, *269*, 382–386, doi:10.1006/bbrc.2000.2310.
